# Ultrasound‐assisted extraction of guava and papaya leaves for the development of functional shrimp patties

**DOI:** 10.1002/fsn3.1706

**Published:** 2020-06-11

**Authors:** Zubda Yaqoob, Muhammad Sajid Arshad, Muhammad Kamran Khan, Muhammad Imran, Muhammad Haseeb Ahmad, Sheraz Ahmad, Mehr un Nisa, Faqir Muhammad Anjum, Urooj Khan, Waseem Khalid, Hafiz Ansar Rasul Suleria

**Affiliations:** ^1^ Institute of Home and Food Sciences Government College University Faisalabad Pakistan; ^2^ Department of Food Science Faculty of Biosciences Cholistan University of Veterinary and Animal Sciences Bahawalpur Pakistan; ^3^ Vice Chancellor Secretariat University of the Gambia Banjul The Gambia; ^4^ Food and Nutritional Sciences The University of Melbourne Melbourne Vic. Australia

**Keywords:** antioxidant assay, functional patties, microbial parameters, physicochemical assay, seafoods

## Abstract

The current study was aimed to evaluate the effects of guava and papaya leaves extract on the antioxidant profile and their outcomes in the storage stability of shrimp patties. Total of seven treatments were prepared by employing guava leaf extract (GLE) and papaya leaf extract (PLE) including control. Both the extracts were employed in the concentration of 1% and 2% each and in combination as 1:1% and 2:2%, respectively. The shrimp patties were kept in ziplock bags at refrigeration temperature, and further analysis was done after 21 days of storage period with intermittent evaluation interval of 7 days. The antioxidant capability of functional shrimp patties was determined by total phenolic content (TPC), 2,2‐diphenyl‐1‐picrylhydrazyl (DPPH), and ferric reducing antioxidant power (FRAP). Higher significant values of TPC, DPPH, and FRAP were observed in the functional shrimp patties enriched with GLE2%:PLE2% at start of the experiment. The physicochemical parameters were observed by hunter color, TVBN, TBARS, and peroxide value (POV). Higher significant values of TVBN, TBARS, and peroxide were observed in the control samples as compared to treatment group GLE2%:PLE2%. The bacterial counts were also higher in control samples as compared to the treatment group GLE2%:PLE2%. The sensorial attributes were observed regarding appearance, taste, texture, odor, and overall acceptability. The maximum scores related all parameters were gathered by control group but significantly lower scores were for the group GLE2%:PLE2%. In conclusion, functional shrimp patties enriched with GLE2%:PLE2% showed better antioxidant capacity, stability, and sensory characteristics during storage.

## Graphical abstract

The current study was aimed to evaluate the effects of guava and papaya leaf extract on the antioxidant profile and their outcomes in the storage stability of shrimp patties.

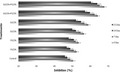

## 1 INTRODUCTION

Shrimps are such species that are increasing their demand at worldwide level. The main reason of their popularity especially in China and Asia is their nutritional composition, that is, amino acids, peptides, and PUFAs in special along with other useful nutrients. They are being referred as one of the vastly developing food products in the global market, possibly because of their apparent nutritional advantages, culinary traits and being a significant source of protein (Ajifolokun, Basson, Osunsanmi, & Zharare, 2018). Their shelf life is limited due to the presence of high moisture content, various degradable compounds, and neutral pH (Viji, Venkateshwarlu, Ravishankar, & Gopal, 2017).

The shelf life of shrimps can be increased by using synthetic as well as natural antioxidants. Presence of synthetic antioxidants play vital role in reducing free radical damage. It also helps to confer antiaging effects. However, at the end level consumers may suffer from their harmful effects, that is, propyl gallate, BHA (butylated hydroxyanisole) and BHT (butylated hydroxyltoluene) (Mona, Isis, Muhammad, & Samir, 2012). On the other hand, natural preservatives such as plant extracts and essential oils are being widely used to replace synthetic preservatives for having admirable antimicrobial and antioxidant potential (Smaldone et al., 2011). They account for lowering the losses by microbial contamination and lipid peroxidation in the food (Erkan, Gunlü, & Genç, 2014).

Guava (*Psidium gujava*) is considered beneficial fruit tree inherent from Mexico. Guava tree is rich in terpenoids, phenolic acids, flavonoids, and tannins (Barbalho et al., 2012; Pérez Gutiénez, Mitchell, & Vargas Solis, 2008); however, the leaves contain mainly tannins, flavonoids, and less amount of terpenoids (Metwally, Omar, Ghazy, & Harraz, 2011). Particularly, the focused area of research is terpenoid content of the leaves and the fruit as well as phenolic contents. Until now, 204 volatile components have been identified in the fruit (Joseph & Priya, 2011). While in the leaves, 86 compounds have been found (Garcia, Quijano, & Pino, 2009). White guava fruit was found to be rich in β‐caryophyllene and β‐bisabolene. Among white and pink fruit forms, variations in the composition have been noticed in the essential oil of fruit (Thuaytong & Anprung, 2011) and of the leaves (Chalannavar, Venugopala, Baijnath, & Odhav, 2014).

Papaya *(Carica papaya)* belongs to family Caricace. It has likewise been accounted for having cancer‐preventing agents and free radical scavenging potential in its leaves separately (Okoko & Ere, 2012). The leaves of papaya contain numerous dynamic parts that can induce antioxidant activity in blood and diminish lipid peroxidation level like paper chymopapain, cyanogenic glycosides, glucosinolates, tocopherol, ascorbic corrosive, and flavonoids along with cystatin (Otsuki et al., 2010). These mixes are identified with mitigating action. *C. Papaya* leaf extracts additionally found to have antibacterial potential (Romasi, Karina, & Parhusip, 2012), antiproliferative to tumor and immunomodulation experiences. It is declared as nonpoisonous in light of the fact that it's LD > 15 g per kg body weight. The leaves additionally contain cardiovascular glycosides, anthraquinones, carpaine, pseudocarpaine, and phenolic mixes (Owoyele, Adebukola, Funmilayo, & Soladoye, 2008). Keeping in view the above facts, the main objective was to minimize the quality loss of shrimp patties during storage as well as enhancing their antioxidant profile with the incorporation of natural extracts of guava and papaya leaves.

## 2 MATERIALS AND METHODS

### 2.1 Procurement of materials and sample preparation

Mature guava and papaya leaves were procured from Ayub Agricultural Research Institute Faisalabad. Frozen shrimps were purchased from supermarket from Faisalabad. All the glassware and chemicals for further testing were used of analytical grade and purchased from Sigma‐Aldrich^®^. Guava and Papaya leaves were sundried for several days and then grounded to fine powder using grinder and sieved to remove woody parts and then stored in air tight bags to avoid moisture gain prior to extraction. Shrimps were thawed and minced, and patties were formed.

### 2.2 Extraction of GLE and PLE

Powdered samples of dried guava and papaya leaves were taken in 500 ml beaker with 70% ethanol at 1:10 solid to solvent ratio. The extraction was carried out employing an ultrasonic probe (VCX‐750, Sonics & Materials). Frequency was set at 20 KHz with 50% amplitude. The extraction was carried out at room temperature for 10 min (Liu, Wang, Lu, & Chiang, 2014).

### 2.3 Filtration and evaporation of solvent

The slurries obtained from two extractions were filtered individually by using Whatman No.1 filter paper. The filtrate of all samples was evaporated using a rotary evaporator attached with a vacuum pump (Model VP18R, Lab tech) and a refrigerated cooling system. Total solvent was dried at 79°C temperature of water bath at a vacuum pressure of 0.07 MPa.

### 2.4 Development of shrimp patties

Minced shrimps were shaped into patties weighing about 5 g each. 6 treatments were given to patties except control as 1% and 2% for GLE, 1% and 2% for PLE, and 1% and 2% in combination for both, respectively. Mincer was used for mincing purpose. 0.5 ml of each solution was added to patties and then stored them in airtight plastic zip lock bags at refrigeration temperature for 21 days. Physiochemical analysis was done at 7‐day intervals.

### 2.5 Total phenolic contents (TPC)

Folin–Ciocalteu method was used to determine the phenolic content of enriched shrimp patties according to Tezcan, Gültekin‐Özgüven, Diken, Özçelik, and Erim (2009). 1 ml of 10% Folin–Ciocalteu reagent was applied to 0.5 ml of a known sample concentration. The mixture was blended and left for 6 min; then, 2 ml of 20% sodium carbonate solution was added in the above mixture. The spectrophotometer (Specord 200/plus, Germany) was used to test the phenols at 760 nm after reacting at 30°C for 60 min. By using standard gallic acid solution, a calibration curve was prepared and the total phenols were expressed as 1 g of equivalent gallic acid (GAE) per gram of sample.

### 2.6 DPPH (2,2‐diphenyl‐1‐picrylhydrazyl) Scavenging Assay

DPPH (2,2‐diphenyl‐1‐picrylhydrazyl) is a stable, highly hued free radical capable of extracting free hydrogen atoms from phenolic antioxidants associated with the formation of colorless hydrazine (DPPH‐H) (Diouf, Stevanovic, & Cloutier, 2009). The radical scavenging potential of GLE‐ and PLE‐enriched shrimp patties was observed by performing method of Mimica‐Dukie, Bozin, Sokovic, & Simin, 2004 with some modifications. 0.1 ml of the homogenized sample was applied to the freshly prepared 0.0012 M DPPH reagent and left in the dark at room temperature for 60 min. Absorption was measured at 517 nm utilizing the spectrophotometer. The each sample was expressed as the percentage of DPPH diminished.

### 2.7 Ferric Reducing Antioxidant Power (FRAP) Assay

The FRAP test was performed on the shrimp patties as per the method described by Benzie and Strain (1996) with few modifications. The FRAP mixture was designed by blending 10 mM 2,4,6‐tri(2‐pyridyl)‐1,3,5‐triazine (TPTZ) in 40 mM HCl; 300 mM acetate buffer (93.6 ml 2 M acetate acid and 6.4 ml 2 M sodium acetate, and pH 3.6,); and 20 mM of ferric chloride in 10:1:1 ratio (v/v/v). Afterward, 200 μl of formulated FRAP chemical compound was added with 50 μl at each test. The blend was placed in the shadow at ambient temperature for 30 min, during which a spectrophotometer was used to measure the absorbance at 593 nm. Different Trolox concentrations (5–50 μg/ml) have been used in the preparation of the standard curve, and the results were measured as (TEAC) mostly in the type of Trolox/gram microgram samples.

### 2.8 Physiochemical analysis of GLE‐ and PLE‐enriched patties

#### 2.8.1 Hunter color (Lab)

The surface color estimations of the GLE‐ and PLE‐enriched shrimp patties were assessed by Hunter colorimeter, with the assistance of measurements that were established according to reference plate (*L* = 89.2, *a* = 0.921, and *b* = 0.783). The CIE L (lightness), CIE a (redness), and CIE b (yellowness) color parameters were estimated by utilizing a mean from 9 arbitrary readings to investigate statistically.

#### 2.8.2 Total Volatile Basic Nitrogen (TVBN)

The TVBN experiment was performed by the method of Malle and Tao (1987) with minor modifications. Samples were blended in a ratio of 1:2 (w/v) with trichloroacetic acid (TCA) and homogenized with blender at speed 2. The solution was then centrifuged at 1008 *g* for 5 min and passed through filter paper Whatman No.1. In the Kjeldahl refining tank, 25 ml of samples were pipetted and 5 ml of 10% sodium hydroxide was applied to them. Steam processing was carried out, and the distillate was titrated against 0.05 M sulfuric acid until the shade turns pink.

#### 2.8.3 Thiobarbituric Reactive Substances (TBARS)

The TBARs value was determined by the method described by Schmedes and Hølmer (1989). 5 g of sample was mixed in a homogenizer with 25 ml of 20 percent trichloroacetic acid (200 g/L) in a solution of 135 ml/L of phosphoric acid for 30 s. The homogenized sample was gone through Whatman filter paper number 4 to dispose of solid particles from the filtrate. 2 ml of 0.02 M TBA solution (3 g/L) was applied in a test tube at that stage with 2 ml filtrate. Samples containing test tubes were incubated at 100°C for 30 min from that point forward and then cooled in running tap water. Using UV‐VIS spectrophotometer, the absorption of supernatant formulations was measured at 532 nm. The TBA values were determined from a standard curve (1,1,3,3‐tetraethoxipropane [TEP]) and expressed as mg malondialdehyde per kilogram (MDA/kg) of sample.

#### 2.8.4 Peroxide Value (POV)

The peroxide value of shrimp enriched with GLE and PLE was measured by using the method outlined by Sallam, Ishioroshi, and Samejima (2004). 3 g sample was collected in the Erlenmeyer flask. To melt the fat in sample, it was heated at 60°C for 3 min in the water bath. After that, to digest the fat, the container was stirred for 3 min with 30 ml of acetic acid–chloroform solution (3:2 v/v). Whatman channel paper number 1 was used to remove the solid particles from the filtrate. The process was accompanied by adding the starch solution as a marker, after the addition of potassium iodide solution (0.5 ml) to filtrate. The titration was carried against the standard sodium thiosulfate solution. POV was calculated by following equation and expressed as milliequivalent peroxide per kilogram of sample.

POV(meq/kg)={(S×N)/W}×100.

where *S* = volume of titrant (ml); *N* = normality of sodium thiosulfate solution (*N* = 0.01); *W* = weight of sample (g).

#### 2.8.5 Total Bacterial Count (TBC)

Total bacterial counts were performed on the outlines given by Linton, Mc Clements, and Patterson (2003) and Karim, Rahman, Muhammad, Zainol, and Ikhwanuddin (2018). A minimum of 10 g sample solution was homogenized with 90 ml of maximum recovery diluent (MRD). Serial dilution has been made before getting desired dilution. Using a sterile glass spreader, 0.1 ml of the dilution was applied to the agar plate. The total count of bacteria was represented as log colony forming units per gram of test samples (log10 CFU g^−1^).

### 2.9 Sensory Evaluation of Shrimp Patties

The shrimp patties were cooked and evaluated by 10 trained panelists using a 9‐point hedonic scale ranging from 9 = like extremely to 1 = dislike extremely (Meilgaard, Civille, & Carr, 2007). All panelists were asked to evaluate appearance, texture, taste, odor, and overall acceptability. The rational assessment was done by giving the mineral water, unsalted crackers, and cups to neutralize their receptors during the sensory evaluation process. The data was recorded during the storage period of 21 days with the interval of 7 days.

### 2.10 Statistical analysis

The data obtained from different parameters were statistically analyzed by using the statistical package, Statistic 8.1, by following the guidelines of Steel and Torrie (2012). The level of significance was observed by using the factorial design under CRD. Three replications were done for each parameter, whereas 10 measurements were recorded for hunter color and 10 panelists were used for sensory evaluation. The means were compared by using least significant differences.

## 3 RESULTS AND DISCUSSIONS

### 3.1 Total phenolic contents

**FIGURE 1:**
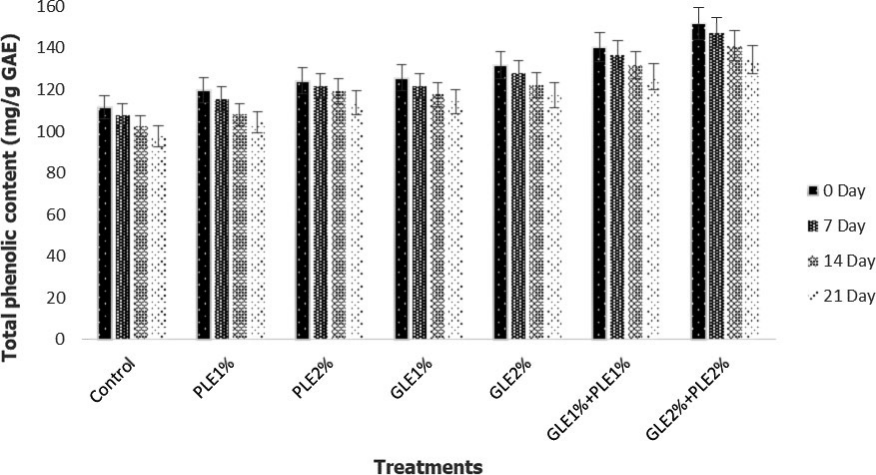
Total phenolic contents (TPC) of PLE‐ and GLE‐enriched functional shrimp patties during storage

Phenolic is one of the main compounds that serve as key antioxidants or free radical terminators. One of the essential criteria for estimating the amount of antioxidants is the calculation of maximum phenolic compounds. Polyphenols demonstrate high antioxidant behavior when plant polyphenols contribute hydrogen atoms to stop fat rancidity by stopping the radical chain reaction through transferring free radicals into more stable atoms. Regarding this manner, they are established as strong antioxidants. The total phenolic content of GLE‐ and PLE‐incorporated shrimp patties is shown in Figure 1. Higher value of total phenolic contents found in functional patties enriched with 2%GLE: 2% PLE at the start of the experiment, whereas minimum value of total phenolic contents was observed in control at day 0. With the passage of time, significant reduction was found in all treatments up to 21 days. The results depicted that higher total phenolic contents was observed in treatment having mixture of 2% GLE and PLE both at day 0 and day 21, whereas minimum contents were found in control. The results are in agreement with the findings of Verma, Rajkumar, Banerjee, Biswas, and Das (2013) who reported that guava leaf powder supplemented in sheep nuggets showed higher antioxidant potential. Similarly, another study reported by Ekaluo, Ikpeme, Ekerette, and Chukwu (2015) showed higher total phenolic contents in treatment with guava leaves than the bitter leaf (*Vernonia amygdalina*). Our results are also supported by the findings of Arshad et al. (2019), who reported that turmeric powder‐treated chicken patties showed higher antioxidant potential. Furthermore, Asghar et al., 2016 depicted that higher phenolic contents were found in papaya leaves than other parts of plant.

### 3.2 2,2‐Diphenyl‐1picrylhydrazyl (DPPH)

**FIGURE 2:**
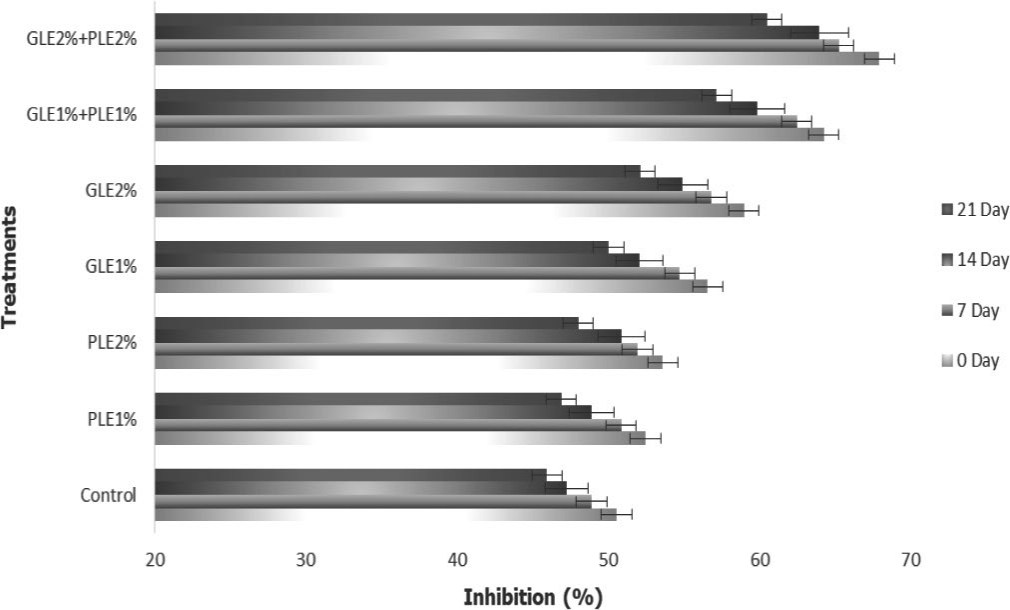
2,2‐Diphenyl‐1picrylhydrazyl (DPPH) of PLE‐ and GLE‐enriched functional shrimp patties during storage

2,2‐Diphenyl‐1‐picrylhydrazyl (DPPH) free radical scavenging approach is a technique to test the antioxidant ability of a drug, sample, or other biological sources. This is the modest approach in which the prospective compound or extract is combined with DPPH solution and absorbance is reported after a specified period of time. DPPH offers the first tactic for assessing the antioxidant potential of a compound, an extract, or other biological sources. This is proved to be the modest method, wherein the prospective compound or extract is mixed with DPPH solution and absorbance is recorded after a defined period. The results regarding the DPPH values of GLE‐ and PLE‐incorporated shrimps patties are shown in Figure 2. Highest DPPH value was observed in the samples treated with GLE2%:PLE2% while the lowest values were shown by the controls at day 0. However, the DPPH values for treated and untreated samples at the end of the storage time were in the range of 45.94 ± 0.13 to 60.49 ± 0.49%. As the time passes, the values of DPPH decreased significantly among all treatments. Controls (45.94 ± 0.13%) were shown to be the lowest, in terms of DPPH inhibition at 21st day of storage while the highest DPPH was shown by the patties treated with GLE2%:PLE2%. The results of our experiment are in agreement with the findings of Fernandes et al. (2018) in which they studied the antioxidant power of spray‐dried guava leaf extract by DPPH assay. Ifesan, Fashakin, Ebosele, and Oyerinde (2013) conducted a study on different plant leaf extract and found papaya leaf hexane extract showed the highest DPPH radical scavenging activity compared with *Anacardium occidentale, Cocos nucifera, Citrus sinesis,* and *Citrus limon*. Arshad, Anjum, et al., 2017; Arshad, Imran, et al., 2017 reported the same synergistic effect of antioxidants from wheat germ oil and alpha‐lipoic acid, induced in the chicken and found the maximum DPPH inhibition in the formulated nuggets.

### 3.3 Ferric reducing antioxidant power

**FIGURE 3:**
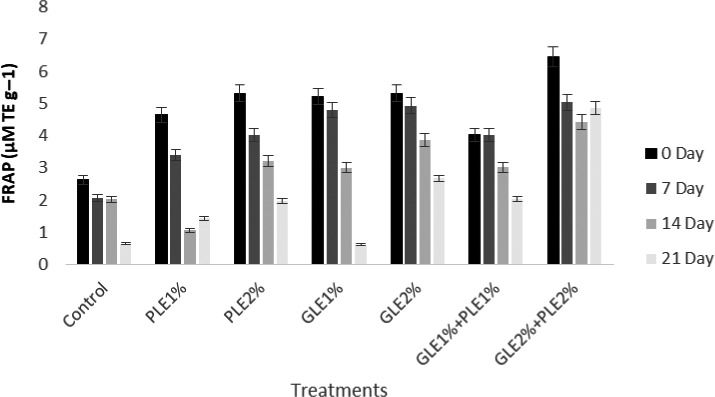
Ferric reducing antioxidant power (FRAP) of PLE‐ and GLE‐enriched functional shrimp patties during storage

One of the most widely used methods for determining the overall antioxidant power of a specimen is the ferric decreasing/antioxidant power (FRAP), which is fairly simple, quick, delicate, and rational to conduct. There are many decentralized studies that have used the FRAP method, and they have produced an extremely huge list of overall antioxidant assessment of a food product that can help in picking diet for greater intakes of antioxidants. Therefore, the FRAP experiment was used to evaluate the bioavailability of antioxidants in food and to investigate the impact of changing environments, processing, handling, and cooking techniques on the overall antioxidant potential of food (Prior, Wu, & Schaich, 2005). The results regarding the FRAP values of GLE‐ and PLE‐enriched shrimp patties are shown in Figure 3. The data showed that the FRAP values were significantly different among all the treatments and the storage time. The results depicted that the FRAP values were in the range of 6.45 ± 0.50 to 2.63 ± 0.43 μM TE/g at 0 day of storage for control and treated samples. The higher values of FRAP was shown by the samples incorporated with GLE2%:PLE2% while the minimum was depicted by the controls at the start of the storage period. The clear inclination was observed that, as the storage time passed, the FRAP values were decreased significantly. Controls showed the minimum FRAP values at the end of the storage time as compared to the patties enriched with GLE2%:PLE2%. Medicinal herbs can be classified into their antioxidant capacity as very low FRAP (<10 µM/100 g), good FRAP (50–100 µM/100 g), high FRAP (100–500 µM/100 g), and very high FRAP (>500 µM/100 g) (Wojdyło, Oszmiański, & Czemerys, 2007). The samples treated with GLE2%:PLE2% (6.45 ± 0.50 μM TE/g) showed the higher FRAP value at start but become lower at the end of storage time but still remained high as compared to the controls which showed the low FRAP (0.65 ± 0.44 μM TE/g). Almost similar values of FRAP were examined by Díaz‐de‐Cerio et al. (2016) in which they quoted the higher antioxidant state of guava leaves at 3.69 ± 0.03 mM FeSO_4_/mg leaf d.w. FRAP. The study conducted by Vuong et al. (2013) formed the evidence of antioxidant and anticancer capacities of saponin‐enriched papaya leaf extracts.

### 3.4 Hunter color

The values regarding color parameters of GLE‐ and PLE‐enriched shrimp patties are shown in the Table 1. The results were showing that the highest L* values were depicted by the samples incorporated with GLE2%:PLE2% while the minimum L* values were seen in the controls at the start of the experiment. With the passage of time, the L* values were decreased in both controls, GLE‐ and PLE‐incorporated samples. At the end of the storage time, the highest L* values were observed by the samples treated with GLE2%:PLE2% while the minimum values were shown by the controls. The less values for L* in the controls were may be due to the melanosis exhibition (Gh et al., 2018). Melanosis is a degradation of phenol compounds and deamination of non‐nitrogenous protein compounds (ManheemBenjakul, Kijroongrojana, & Visessanguan, 2012). The effect of natural plant extracts on melanosis inhibition was previously reported by (Gokoglu & Yerlikaya, 2018). Alparsalan et al. (2016) have reported the inhibition of color changes in shrimp samples by orange essential oil coating. Yatmaz and Gokoglu (2016) have reported the melanosis inhibition by application of plant extract–sulfide combination on shrimps. The decrease in L* values in the chicken nuggets by inducing the quercetin and alpha‐tocopherol was also stated by Sohaib et al. (2017).

**TABLE 1:** Hunter color of PLE‐ and GLE‐enriched functional shrimp patties during storage

Hunter Color (Lab)					
Parameters	Treatments	0 day	7 days	14 days	21 days
*L* ^*^	Control	44.50 ± 0.42dw	41.64 ± 0.33dx	38.10 ± 0.45cy	35.60 ± 0.42dz
	PLE 1%	46.79 ± 0.33cw	44.50 ± 0.27dx	39.68 ± 0.40cy	42.33 ± 0.26cx
	PLE 2%	47.06 ± 0.20cw	44.53 ± 0.37dx	38.97 ± 0.40cy	42.37 ± 0.26cx
	GLE 1%	47.92 ± 0.21cw	45.81 ± 0.37cx	44.29 ± 0.26bx	40.28 ± 0.31cz
	GLE 2%	49.92 ± 0.43aw	46.02 ± 0.35bx	43.31 ± 0.17by	44.01 ± 0.31by
	GLE1% + PLE1%	48.33 ± 0.41bw	47.37 ± 0.21aw	45.08 ± 0.34ax	44.41 ± 0.32bx
	GLE2% + PLE2%	49.83 ± 0.51aw	46.81 ± 0.23bx	45.20 ± 0.25ax	45.02 ± 0.39ax
	Mean	47.76 ± 0.26a	45.24 ± 0.24b	42.09 ± 0.29c	42.00 ± 0.34c
*a* ^*^	Control	0.20 ± 0.02ez	1.02 ± 0.14ey	1.39 ± 0.06cx	2.27 ± 0.35dw
	PLE 1%	0.29 ± 0.01cz	1.16 ± 0.37ey	2.67 ± 0.16bw	2.41 ± 0.02dx
	PLE 2%	0.25 ± 0.03dz	1.32 ± 0.14dy	2.88 ± 0.18bx	3.55 ± 0.22cw
	GLE 1%	0.31 ± 0.03cz	1.46 ± 0.17dy	2.77 ± 0.20bx	3.32 ± 0.31cw
	GLE 2%	0.23 ± 0.01ez	2.48 ± 0.14cy	2.89 ± 0.20bx	3.71 ± 0.20cw
	GLE1% + PLE1%	0.38 ± 0.02bz	2.64 ± 0.25by	3.75 ± 0.26ax	4.09 ± 0.12bw
	GLE2% + PLE2%	0.46 ± 0.01az	2.71 ± 0.30ay	3.87 ± 0.32ax	4.67 ± 0.28aw
	Mean	0.30 ± 0.03d	1.83 ± 0.11c	2.89 ± 0.12b	3.43 ± 0.21a
*b* ^*^	Control	1.93 ± 0.01dz	1.97 ± 0.02ey	3.03 ± 0.01dx	4.08 ± 0.01dw
	PLE 1%	1.35 ± 0.03cz	2.09 ± 0.01dy	3.12 ± 0.10dx	5.82 ± 1.02cw
	PLE 2%	1.38 ± 0.01cz	2.68 ± 0.03cy	3.37 ± 0.32cx	6.02 ± 1.01cw
	GLE 1%	1.90 ± 0.02bz	2.97 ± 0.31cy	3.97 ± 0.36bx	8.29 ± 0.03aw
	GLE 2%	1.97 ± 0.02bz	3.02 ± 0.27by	4.08 ± 0.41bx	8.93 ± 1.00aw
	GLE1% + PLE1%	2.70 ± 0.40az	3.18 ± 0.43by	4.92 ± 0.43ax	7.78 ± 1.18bw
	GLE2% + PLE2%	2.88 ± 0.41az	3.39 ± 0.17ay	5.01 ± 0.37ax	9.89 ± 0.44aw
	Mean	2.02 ± 0.08d	2.76 ± 0.14c	3.93 ± 0.09b	7.26 ± 0.24a

Note

The values are mean ± *SD* of ten independent determinations. Means carrying different letters in a column differed significantly (*p* < .05).

Abbreviations: GLE, guava leaf extract; PLE, papaya leaf extract.

^a‐e^Values with different letter within a column are significantly different (*p* < .05).

^w‐z^Values with different letter within a row are significantly different (*p* < .05).

The results regarding a* (redness) values for GLE‐ and PLE‐incorporated shrimp patties were shown in Table 1. Higher a* value was observed in the samples treated with GLE2%:PLE2% at the 0 day of storage while the minimum was seen in controls. The a* value was also increased throughout the storage period but the shrimp patties incorporated with GLE2%:PLE2% showed the higher values. On the other hand, the controls showed the minimum a* values at the end of storage period. The redness increased from light to bright red during storage in OLEO applied shrimps (Alparsalan et al. 2016). Arshad et al. (2019) reported the increase of redness in turmeric powder applied chicken meat.

The results regarding b* (yellowness) values of GLE‐ and PLE‐incorporated shrimp patties are shown in the Table 1. The data obtained were shown that b* values for control and GLE and PLE applied samples were in the range of 2.88 ± 0.41 to 1.97 ± 0.01 and 9.89 ± 0.44 to 4.08 ± 0.01 at the 0 and 21st days of storage, respectively. The minimum b* values were observed in control samples at 0 day of storage while the maximum yellowness was observed in the GLE2%:PLE2% enriched samples. With the passage of time, the yellowness increased in both treated and untreated samples but remained significantly different among the treatments. The peak b* values were seen in GLE2%:PLE2% incorporated samples while the lowest values were observed in control samples (4.08 ± 0.01) at the end of the storage interval. The results are in agreement with the findings of Kim, Choi, et al. (2013) who reported that the yellowness (b*) increased in beef patties supplemented with soy sauce as source of antioxidant potential with the increase in storage interval. Furthermore, Kim, Jin, et al. (2013) depicted that with the addition of antioxidant source in patties increased the yellowness (b*) which is in line with the outcomes of present study.

### 3.5 Total volatile basic nitrogen

Microbiological spoilage causes the formation of volatile bases (mainly ammonia, dimethylamine, and trimethylamine) during the postmortem storage of a wide variety of seafood, which have often been determined to indirectly measure the freshness quality of such seafood. Total volatile nitrogen is almost exclusively composed of ammonia. When ammonia production increases during spoilage due to protein deamination, its assessment is an easy way to assess seafood value (Howgate, 2010). The results regarding the TVBN content of GLE‐ and PLE‐enriched shrimp patties were shown in the Table 2. The maximum value of TVBN was observed in the control samples at 0 day while the minimum values were shown by the samples treated with GLE 2%: PLE 2% followed by GLE 2%. As the storage time increased, the TVBN values also increased and a significant rise was observed in control between 14th and 21st days of storage. Whereas the samples treated with GLE2%:PLE2% have shown the suspending effect on TVBN values and reached to the maximum extent of 17.43 ± 0.31 mg Nitrogen/100 g as compared to other treatments. TVBN is commonly used as a quality index for shrimps in Australia and Japan, setting a 30 mg/100 g acceptability limit for commercial purposes (Smaldone et al., 2011). However, results from this study showed that the samples treated with GLE and PLE still had their final TVBN within the acceptable limits, since they all had values less than 30 mg N/100 g. Our results correspond with the study of Alparslan et al. (2016), who observed the moderate increase of TVBN values in orange peel essential oil coated shrimps as compared to the control samples. When bacterial counts were rather small, the early rise in TVBN content in control samples suggested that autolytic processes result in the development of volatile bases (Finne, 1982). On the other hand, the decrease in the TVBN in GLE2%:PLE2% treated shrimp patties was the indication of the presence of the strong effect of antioxidants in GLE and PLE, which prevented the spoilage bacteria during storage (Biswas, Rogers, McLaughlin, Daniels, & Yadav, 2013; Nirosha & Mangalanayaki, 2013). Khan et al., 2015 also found the increase in TVBN content in untreated samples of rohu fish but reported the significant delaying of TVBN by whey protein edible coating on them.

**TABLE 2:** Total volatile basic nitrogen (TVBN) and total bacterial count of PLE‐ and GLE‐enriched functional shrimp patties during storage

Treatments	TVBN (mg Nitrogen/100 g)				Total Bacterial Count (CFU/g)			
	0 day	7 days	14 days	21 days	0 day	7 days	14 days	21 days
Control	13.12 ± 0.32az	19.01 ± 0.32ay	27.05 ± 1.02ax	43.05 ± 0.40aw	5.37 ± 0.41az	7.48 ± 0.40ay	7.60 ± 0.50ax	8.56 ± 0.30aw
PLE1%	9.48 ± 0.45bz	17.58 ± 0.31by	22.63 ± 0.41bx	28.66 ± 0.67bw	4.55 ± 0.30bz	4.46 ± 0.40cy	6.45 ± 0.47bx	7.63 ± 0.26bw
PLE2%	8.27 ± 0.40cz	17.01 ± 0.13by	21.97 ± 0.41bx	22.45 ± 0.41cw	3.61 ± 0.31cz	4.97 ± 0.42by	5.38 ± 0.29cx	6.55 ± 0.39cw
GLE1%	6.50 ± 0.10dz	13.59 ± 0.47cy	17.61 ± 0.31cx	19.55 ± 0.26dw	3.30 ± 0.30cz	4.64 ± 0.42cy	5.32 ± 0.21cx	6.10 ± 0.31cw
GLE2%	5.05 ± 0.50dz	11.61 ± 0.38dy	15.65 ± 0.31dx	18.12 ± 0.31dw	2.64 ± 0.22dz	3.91 ± 0.20dy	4.27 ± 0.41ex	5.71 ± 0.34dw
GLE1% + PLE1%	5.23 ± 0.60dz	12.44 ± 0.43dy	16.01 ± 0.31cx	19.26 ± 0.36dw	3.11 ± 0.30cz	4.02 ± 0.34dy	4.91 ± 0.42dx	6.21 ± 0.33cw
GLE2% + PLE2%	4.76 ± 1.00ez	11.47 ± 0.43dy	16.25 ± 0.17cx	17.43 ± 0.31ew	2.16 ± 0.27dz	3.74 ± 0.20ey	4.73 ± 0.42x	5.01 ± 0.30ew
Mean	7.49 ± 0.41d	14.67 ± 0.12c	19.60 ± 0.16b	24.07 ± 0.21a	3.53 ± 0.36d	4.75 ± 0.29c	5.52 ± 0.41b	6.54 ± 0.19a

Note

The values are mean ± *SD* of three independent determinations. Means carrying different letters in a column differed significantly (*p* < .05).

Abbreviations: GLE, guava leaf extract; PLE, papaya leaf extract.

^a‐e^Values with different letter within a column are significantly different (*p* < .05).

^w‐z^Values with different letter within a row are significantly different (*p* < .05).

### 3.6 Total bacterial counts

The total number of bacteria is one of the key indicators of hygiene control in food processing. The total bacterial counts of GLE‐ and PLE‐supplemented shrimp patties are shown in Table 2. The results depicted that the bacterial count was higher in control samples at the start of the experiment but was minimum in GLE2%:PLE2% enriched shrimp patties. Bacterial count was enhanced among all the treatments but significantly reduced in GLE2%:PLE2% and GLE 2% treated samples which have shown the minimum values (5.01 ± 0.30 CFU/g) and (5.71 ± 0.34 CFU/g) at the 21st day of storage. Al‐dagal and Bazaraa (1999), found that the sustainable limit of aerobic plate count was 6 log10 CFU g^−1^, according to which, the GLE2%:PLE2% enriched patties was under the acceptable limit. Our results are in agreement with the results found by Kavimandan and Saraf (2016) that determined the antibacterial activity of PLE against E. coli, and with another study carried out by Mailoa et al. (2014) who observed that the antimicrobial activities of GLE were considerably due to it is rich in tannins.

### 3.7 Thiobarbituric Acid Reactive Substances (TBARS)

TBARS is most probably the oldest and one of the most commonly employed methods to measure lipid peroxidation end product malondialdehyde, a volatile aldehyde produced by polyunsaturated fatty acid lipid peroxidation. The statistical results regarding TBARS value of GLE and PLE enriched functional patties with different storage intervals are given in Table 3. The results showed that higher TBARS value was observed in the control, whereas minimum was found in shrimp patties enriched with GLE 2%:PLE 2% at day 0. As the time increased, the TBARS values were also increased significantly in control followed by PLE 2% while the minimum value was exhibited by the patties treated with GLE 2%:PLE 2%. The total amount of 2 mg malondialdehyde/kg TBARS is taken in the test sample as an acceptable limit in frozen or dried goods, according to the National Iranian Standards Organization (Gh et al., 2018). The consequences of this examination likewise demonstrated that with the expansion of storage period, the measure of TBARS was expanded, but the values in all treatments were remained in acceptable limits during that specified period. The minimum content of TBA in shrimp patties can be due to the use of GLE and PLE which have phenolic and flavonoid content in a larger amounts (Karim et al., 2018) (Vuong et al., 2013). On the other hand, the high content of TBARS in control samples can be due to the lipid peroxidation (Alparslan et al. 2016). The quality changes in the patties enriched with 2%GLE:2%PLE followed by 2%GLE were lower than other treatments. Our results showed the resemblance with the findings of Yu et al. (2018) in which he reported the gradual increase in TBARS value with storage time; however, the values remained below the maximum limit (2 mg MDA/kg). Das, Rajkumar, Verma, and Swarup (2012) showed that the *Moringa oleiferia* leaves concentrate was more viable than BHT for anticipating expanded TBARS number of precooked goat meat patties during storage. Arshad, Anjum, et al. (2017) formulated a study on the manipulation of alpha‐lipoic acid and wheat germ oil in broiler diet and found to have lower TBARS value in the nuggets during storage. Nisar, Arshad, Yasin, Arshad, and Nadeem (2019) found the same trend of slowing down the formation of TBARS in chicken meat by incorporation of moringa leaf powder.

**TABLE 3:** Thiobarbituric acid reactive substances (TBARS) and peroxide value (POV) of PLE‐ and GLE‐enriched functional shrimp patties during storage

Treatments	TBARS (MDA/kg)				POV(meq peroxide/kg)			
	0	7	14	21	0	7	14	21
Control	0.31 ± 0.03az	0.34 ± 0.03ay	0.39 ± 0.03ax	0.46 ± 0.03aw	0.23 ± 0.01az	0.27 ± 0.03ay	0.33 ± 0.03ax	0.39 ± 0.03aw
PLE1%	0.27 ± 0.02bz	0.32 ± 0.02by	0.36 ± 0.04bx	0.41 ± 0.04bw	0.21 ± 0.03bz	0.25 ± 0.02by	0.32 ± 0.02ax	0.37 ± 0.04bw
PLE2%	0.26 ± 0.02bz	0.3 ± 0.03by	0.36 ± 0.03bx	0.42 ± 0.03bw	0.2 ± 0.02bz	0.25 ± 0.04by	0.29 ± 0.02bx	0.35 ± 0.03cw
GLE1%	0.24 ± 0.01cz	0.29 ± 0.02cy	0.34 ± 0.03cx	0.39 ± 0.02cw	0.18 ± 0.01cz	0.22 ± 0.03cy	0.27 ± 0.03bx	0.32 ± 0.01dw
GLE2%	0.21 ± 0.02dz	0.26 ± 0.01dy	0.33 ± 0.04cx	0.36 ± 0.03cw	0.16 ± 0.02dz	0.21 ± 0.01cy	0.25 ± 0.03cx	0.31 ± 0.03dw
GLE1% + PLE1%	0.23 ± 0.03cz	0.26 ± 0.03dy	0.32 ± 0.02cx	0.37 ± 0.03cw	0.17 ± 0.02cz	0.21 ± 0.03cy	0.26 ± 0.04bx	0.31 ± 0.02dw
GLE2% + PLE2%	0.19 ± 0.01ez	0.23 ± 0.01ey	0.26 ± 0.02dx	0.31 ± 0.02dw	0.14 ± 0.01ez	0.18 ± 0.01dy	0.22 ± 0.01dx	0.26 ± 0.01ew
Mean	0.24 ± 0.02d	0.29 ± 0.03c	0.34 ± 0.02b	0.39 ± 0.03a	0.18 ± 0.01d	0.23 ± 0.01c	0.28 ± 0.03b	0.33 ± 0.02a

Note

The values are mean ± *SD* of three independent determinations. Means carrying different letters in a column differed significantly (*p* < .05).

Abbreviations: GLE, guava leaf extract; PLE, papaya leaf extract.

^a‐e^Values with different letter within a column are significantly different (*p* < .05).

^w‐z^Values with different letter within a row are significantly different (*p* < .05).

### 3.8 Peroxide value

The POV is the indicator of lipid peroxidation in the meat samples (Domínguez et al., 2019). The results regarding the POV of enriched shrimp patties are shown in Table 3. Higher POV was showed by control samples, while the lowest values were revealed by the patties enriched with GLE2%:PLE2% at the start of research. As like as TBARS, the POV were also intensified with the storage intervals, the highest POV was seen in the control followed by PLE 1% while the minimum value was shown by shrimp patties treated with 2%GLE:2% PLE at the 21st day of storage. As in our study, the POV in control was higher both at the start and end of the experiment. The POV was observed to be significantly decreased in the patties treated with 2%GLE:2%PLE as compared to all treatments because of the combination of antioxidants provided by both GLE and PLE extracts (Karim et al., 2018; Patil, Patil, Patil, & Patil, 2014). Arshad et al. (2019) reported the effects of combined treatment of turmeric powder and irradiations in chicken meat and observed the increase of peroxide value with the extension of storage time but the production rate was lower in turmeric treated samples which also concur with our study. Khan et al., 2015 also conducted a study on the effects of whey protein edible coating on rohu fish and supported the increase of POV in all treatments but also reported the less increase of POV in treated samples. Sofi, Raju, Lakshmisha, & Singh, 2016 presented the benefits of papaya seed extracts in delaying the peroxide value in fish.

### 3.9 Sensory evaluation of GLE and PLE enriched shrimp patties

Sensory characteristics of a food product are very important in consumer appeal. They play a vital role in the consumer demand toward that product (Sharif, Masoos, Hafiz, & Muhammmad, 2017). The sensory evaluation of GLE‐ and PLE‐enriched shrimp patties was carried by trained panelists. The mean score for appearance, texture, taste, odor, and overall acceptability of functional shrimp patties enriched with GLE and PLE is presented in Table 4. The results showed that the sensory attributes were decreased with the passage of storage time. The results showed that, with the increase in the concentration of the extracts applied, the sensory attributes decreased slightly but remained in the acceptable stage throughout the storage period which is correlated with the results of lightness which also increased due to increase the amount of extracts applied. The results regarding the odor also directly related with the results of TBARS and POV. The decreasing trend was found in odor as well as in TBARS and POV as the GLE and PLE extracts applied. The findings of our research are concurring with the results of Alparslan et al. (2016), who similarly found that the application of essential oil extracted from orange leaf, improved the sensory attributes of shrimps during storage. Verma et al., 2013 reported the positive effect of guava leaf powder on sensory characteristics of cooked sheep meat nuggets. Moreover, Udayasoorian, Peter, Sabina, Indumathi, and Muthusamy (2017) have found the similar trend toward the color and odor of shrimps treated with natural extracts.

**TABLE 4:** Sensory Evaluation of PLE‐ and GLE‐enriched functional shrimp patties during storage

Sensory Evaluation					
Parameters	Treatments	0	7	14	21	
Appearance	Control	7.3 ± 0.24bw	7.2 ± 0.22aw	7.0 ± 0.24ax	6.9 ± 0.20ax	
	PLE1%	7.3 ± 0.23bw	7.1 ± 0.24ax	7.0 ± 0.28ay	6.9 ± 0.23ay	
	PLE2%	7.5 ± 0.08aw	6.8 ± 0.65bx	6.7 ± 0.61bx	6.6 ± 0.19by	
	GLE1%	7.3 ± 0.09bw	6.6 ± 0.42cx	6.5 ± 0.62bx	6.4 ± 0.21by	
	GLE2%	7.2 ± 0.21bw	6.7 ± 0.40cx	6.4 ± 0.31cy	6.2 ± 0.15cz	
	GLE1% + PLE1%	7.3 ± 0.23bw	6.5 ± 0.38dx	6.4 ± 0.33cx	6.3 ± 0.15cy	
	GLE2% + PLE2%	7.2 ± 0.20bw	6.4 ± 0.31dx	6.3 ± 0.28cx	6.1 ± 0.21cy	
	Mean	7.3 ± 0.36a	6.76 ± 0.52b	6.61 ± 0.49c	6.49 ± 0.39d	
Texture	Control	7.4 ± 0.12aw	7.3 ± 0.63ax	7.1 ± 0.62ay	6.8 ± 0.55az	
	PLE1%	7.3 ± 0.13bw	7.2 ± 0.64bw	6.9 ± 0.61bx	6.7 ± 0.51by	
	PLE2%	7.3 ± 0.18bw	7.2 ± 0.67bw	6.7 ± 0.62cx	6.5 ± 0.48by	
	GLE1%	7.5 ± 0.32aw	7.4 ± 0.03aw	7.3 ± 0.59ax	6.9 ± 0.45ay	
	GLE2%	7.2 ± 0.25cw	7.1 ± 0.62cw	6.8 ± 0.55bx	6.6 ± 0.39by	
	GLE1% + PLE1%	6.9 ± 0.21dw	6.8 ± 0.22cw	6.5 ± 0.52cx	6.4 ± 0.35by	
	GLE2% + PLE2%	6.5 ± 0.23ew	6.4 ± 0.22dw	6.2 ± 0.49dx	6.1 ± 0.30cy	
	Mean	7.16 ± 0.56a	7.06 ± 0.36b	6.79 ± 0.46c	6.57 ± 0.51d	
Taste	Control	7.4 ± 0.27aw	7.2 ± 0.03ax	6.9 ± 0.10ay	6.7 ± 0.11az	
	PLE1%	7.4 ± 0.38aw	7.2 ± 0.02ax	6.7 ± 0.17by	6.5 ± 0.13bz	
	PLE2%	7.3 ± 0.32bw	7.1 ± 0.01ax	6.7 ± 0.16by	6.4 ± 0.21bz	
	GLE1%	6.5 ± 0.46cw	6.4 ± 0.03bw	6.3 ± 0.19cx	6.1 ± 0.23cy	
	GLE2%	6.5 ± 0.44cw	6.3 ± 0.01bw	6.1 ± 0.15cx	5.7 ± 0.13dy	
	GLE1% + PLE1%	6.4 ± 0.41cw	6.1 ± 0.13cx	5.9 ± 0.20dy	5.5 ± 0.21dz	
	GLE2% + PLE2%	6.3 ± 0.31cw	6.1 ± 0.12cx	5.7 ± 0.21dy	5.4 ± 0.26dz	
	Mean	6.83 ± 0.36a	6.63 ± 0.42b	6.33 ± 0.36c	6.04 ± 0.36d	
Odor	Control	7.5 ± 0.01aw	6.7 ± 0.21ax	6.2 ± 0.57ay	6.0 ± 0.21az	
	PLE1%	7.5 ± 0.12aw	6.6 ± 0.25ax	6.1 ± 0.52ay	6.0 ± 0.23az	
	PLE2%	7.1 ± 0.3bw	6.6 ± 0.25ax	6.3 ± 0.21ay	6.01 ± 0.51az	
	GLE1%	6.3 ± 0.04cw	6.4 ± 0.02bw	6.3 ± 0.23ax	5.5 ± 0.36by	
	GLE2%	6.2 ± 0.05cw	6.1 ± 0.01bw	5.9 ± 0.12bx	5.7 ± 0.50by	
	GLE1% + PLE1%	6.1 ± 0.18cw	5.9 ± 0.10cx	5.9 ± 0.26bx	5.6 ± 0.30by	
	GLE2% + PLE2%	5.9 ± 0.19cw	5.71 ± 0.12cx	5.6 ± 0.72by	5.3 ± 0.21cz	
	Mean	6.66 ± 0.23a	6.29 ± 0.32b	6.04 ± 0.16c	5.74 ± 0.41d	
Overall Acceptability	Control	7.43 ± 0.33aw	7.10 ± 0.07ax	6.4 ± 0.30ay	6.2 ± 0.03az	
	PLE1%	7.40 ± 0.16aw	7.02 ± 0.07ax	6.25 ± 0.02ay	6.23 ± 0.04ay	
	PLE2%	7.46 ± 0.06aw	6.8 ± 0.09bx	6.13 ± 0.51by	6.01 ± 0.51bz	
	GLE1%	6.95 ± 0.69bw	6.8 ± 0.07bx	5.96 ± 0.13cy	5.83 ± 0.06bz	
	GLE2%	6.86 ± 0.07bw	6.61 ± 0.12cx	5.86 ± 0.06cy	5.71 ± 0.12cz	
	GLE1% + PLE1%	6.46 ± 0.41bw	6.59 ± 0.17cx	5.89 ± 0.06cy	5.62 ± 0.31cz	
	GLE2% + PLE2%	6.39 ± 0.42bw	6.01 ± 0.13dx	5.6 ± 0.03dy	5.4 ± 0.30cz	
	Mean	6.99 ± 0.19a	6.70 ± 0.18b	6.01 ± 0.22c	5.86 ± 0.36d	

Note

The values are mean ± *SD* of ten independent determinations. Means carrying different letters in a column differed significantly (*p* < .05).

Abbreviations: GLE, guava leaf extract; PLE, papaya leaf extract.

^a‐e^Values with different letter within a column are significantly different (*p* < .05).

^w‐z^Values with different letter within a row are significantly different (*p* < .05).

## 4 CONCLUSIONS

This study revealed the significance of GLE and PLE on the storage stability and antioxidant activity of shrimp patties during storage. The antioxidant potential of shrimp patties was found to significantly higher in the treatments with combination of GLE (2%) and PLE (2%), whereas lower was observed in control. The stability parameters showed that TBARS and peroxide value were found to be higher in control as compared to the other treatments during 21 days of storage. Total aerobic counts were also found to be higher in control, and minimum was observed in treatments with the combination of GLE (2%) and PLE (2%). The sensory score given by the panelist was found to be higher in the control as compared to the other treatments but found acceptable score in other treatments. It is concluded that the shrimp patties made by the combination of GLE and PLE found significantly better results regarding the physical, microbiological, and antioxidant profile during storage.

## CONFLICT OF INTERESTS

The authors declare no conflict of interest.

## ETHICAL APPROVAL

This study has nothing to do with human and animal testing.

## DECLARATION

The authors did not use the human subjects.

## References

[fsn31706-bib-0001] Ajifolokun, O. M. , Basson, A. K. , Osunsanmi, F. O. , & Zharare, G. E. (2018). Effects of drying methods on quality attributes of shrimps. Journal of Food Processing and Technology, 10(772), 2.

[fsn31706-bib-0002] Al‐dagal, M. M. , & Bazaraa, W. A. (1999). Extension of shelf life of whole and peeled shrimp with organic acid salts and bifidobacteria. Journal of Food Protection, 62(1), 51–56. 10.4315/0362-028X-62.1.51 9921829

[fsn31706-bib-0003] Alparslan, Y. , Yapıcı, H. H. , Metin, C. , Baygar, T. , Günlü, A. , & Baygar, T. (2016). Quality assessment of shrimps preserved with orange leaf essential oil incorporated gelatin. LWT‐Food Science and Technology, 72, 457–466.

[fsn31706-bib-0004] Arshad, M. S. , Amjad, Z. , Yasin, M. , Saeed, F. , Imran, A. , Sohaib, M. , … Hussain, S. (2019). Quality and stability evaluation of chicken meat treated with gamma irradiation and turmeric powder. International Journal of Food Properties, 22(1), 154–172.

[fsn31706-bib-0005] Arshad, M. S. , Anjum, F. M. , Khan, M. I. , Saeed, F. , Imran, A. , Sohaib, M. , … Hussain, S. (2017). Manipulation of natural antioxidants in feed to enhance the oxidative stability and quality of broiler breast meat and nuggets. Journal of Food Processing and Preservation, 41(1), e12849 10.1111/jfpp.12849

[fsn31706-bib-0006] Arshad, M. S. , Imran, A. , Nadeem, M. T. , Sohaib, M. , Saeed, F. , Anjum, F. M. , … Hussain, S. (2017). Enhancing the quality and lipid stability of chicken nuggets using natural antioxidants. Lipids in Health and Disease, 16(1), 108 10.1186/s12944-017-0496-4 28595582 PMC5465442

[fsn31706-bib-0007] Asghar, N. , Naqvi, S. A. R. , Hussain, Z. , Rasool, N. , Khan, Z. A. , Shahzad, S. A. , … Jaafar, H. Z. (2016). Compositional difference in antioxidant and antibacterial activity of all parts of the *Carica papaya* using different solvents. Chemistry Central Journal, 10(1), 5 10.1186/s13065-016-0149-0 26848308 PMC4741006

[fsn31706-bib-0008] Barbalho, S. M. , Farinazzi‐Machado, F. M. V. , de Alvares Goulalt, R. , Saad Bnmnati, A. C. , Ottoboni, M. B. , Machado Bueno Ottoboni, A. M. , & Teixeira Nicolau, C. C. (2012). *Psidium Guajava* (Guava): A plant of multipmpose medicinal applications. Medicinal and Aromatic Plants, 1, 1–6.

[fsn31706-bib-0009] Benzie, I. F. , & Strain, J. J. (1996). The ferric reducing ability of plasma (FRAP) as a measure of “antioxidant power”: The FRAP assay. Analytical Bio‐chem., 239(1), 70–76. 10.1006/abio.1996.0292 8660627

[fsn31706-bib-0010] Biswas, B. , Rogers, K. , McLaughlin, F. , Daniels, D. , & Yadav, A. (2013). Antimicrobial activities of leaf extracts of guava (*Psidium guajava* L.) on two gram‐negative and gram‐positive bacteria. International Journal of Micro‐biology, 2013:1–7. 10.1155/2013/746165 PMC3817707 24223039

[fsn31706-bib-0011] Chalannavar, R. K. , Venugopala, K. N. , Baijnath, H. , & Odhav, B. (2014). The chemical composition of leaf essential oils of *Psidium guajava* L (white and pink fruit forms) from South Africa. Journal of Essential Oil Bearing Plants, 17(6), 1293–1302.

[fsn31706-bib-0012] Das, A. K. , Rajkumar, V. , Verma, A. K. , & Swarup, D. (2012). *Moringa oleiferia* leaf extract: A natural antioxidant for retarding lipid peroxidation in cooked goat meat patties. International Journal of Food Science & Technology, 47(3), 585–591. 10.1111/j.1365-2621.2011.02881.x

[fsn31706-bib-0013] Díaz‐de‐Cerio, E. , Verardo, V. , Gómez‐Caravaca, A. , Fernández‐Gutiérrez, A. , & Segura‐Carretero, A. (2016). Exploratory characterization of phenolic compounds with demonstrated anti‐diabetic activity in guava leaves at different Oxidation States. International Journal of Molecular Sciences, 17(5), 699 10.3390/ijms17050699 PMC4881523 27187352

[fsn31706-bib-0014] Diouf, P. N. , Stevanovic, T. , & Cloutier, A. (2009). Study on chemical composition, antioxidant and anti‐inflammatory activities of hot water extract from *Picea mariana* bark and its proanthocyanidin‐rich fractions. Food Chemistry, 113, 897–902.

[fsn31706-bib-0015] Domínguez, R. , Pateiro, M. , Gagaoua, M. , Barba, F. J. , Zhang, W. , & Lorenzo, J. M. (2019). A comprehensive review on lipid oxidation in meat and meat products. Antioxidants, 8(10), 429 10.3390/antiox8100429 PMC6827023 31557858

[fsn31706-bib-0016] Ekaluo, U. B. , Ikpeme, E. V. , Ekerette, E. E. , & Chukwu, C. I. (2015). In vitro antioxidant and free radical activity of some Nigerian medicinal plants: Bitter leaf (*Vernonia amygdalina* L.) and guava (*Psidium guajava*. Del.). Research Journal of Medicinal Plant, 9(5), 215–226.

[fsn31706-bib-0017] Erkan, N. , Gunlü, A. , & Genç, İ. Y. (2014). Alternative seafood preservation technologies: ionizing radiation and high pressure processing. Journal of FisheriesSciences. com, 8(3), 238.

[fsn31706-bib-0018] Fernandes, M. R. , Kabeya, L. M. , Souza, C. R. , Massarioli, A. P. , Alencar, S. M. , & Oliveira, W. P. (2018). Antioxidant activity of spray‐dried extracts of *Psidium guajava* leaves. Journal of Food Research, 7(4), 141–148. 10.5539/jfr.v7n4p141

[fsn31706-bib-0019] Finne, G. (1982). Enzymatic ammonia production in penaeid shrimp held on ice. Chemistry and Biochemistry of Marine Food Products, 323–331.

[fsn31706-bib-0020] Garcia, M. , Quijano, C. E. , & Pino, J. A. (2009). Free and glycol‐sidically bound volatiles in guava leaves (*Psidium guajava* L.) ICA‐I cultivar. Journal of Essential Oil Research, 21, 131–134.

[fsn31706-bib-0021] Gh, Z. , Etemadian, Y. , Khanipour, A. A. , & Fahim, A. (2018). The quality changes of frozen and dried tiny shrimp (*Macrobrachium nipponense*) meat during six months storage. Journal of Nutrition and Health Sciences, 5(2), 209 10.15744/2393-9060.5.206

[fsn31706-bib-0022] Gokoglu, N. , & Yerlikaya, P. (2008). Inhibition effects of grape seed extracts on melanosis formation in shrimp (Parapenaeus longirostris). International journal of food science & technology, 43(6), 1004–1008.

[fsn31706-bib-0024] Howgate, P. (2010). A critical review of total volatile bases and trimethylamine as indices of freshness of fish. Part 1. Determination. Electronic Journal of Environmental, Agricultural & Food Chemistry, 9(1), 29–57.

[fsn31706-bib-0025] Ifesan, B. O. T. , Fashakin, J. F. , Ebosele, F. , & Oyerinde, A. S. (2013). Antioxidant and antimicrobial properties of selected plant leaves. European Journal of Medicinal Plants, 3, 465–473. 10.9734/EJMP/2013/3383

[fsn31706-bib-0027] Joseph, B. , & Priya, R. M. (2011). Phytochemical and biopharmaceutical aspects of *Psidium guajava* L. essential oil: A review. Research Journal of Medicinal Plant, 5, 432–442.

[fsn31706-bib-0029] Karim, N. U. , Rahman, I. R. A. , Muhammad, M. K. , Zainol, N. A. K. , & Ikhwanuddin, M. (2018). Effects of Guava, *Psidium guajava* leaves extract coating on giant freshwater prawns, *Macrobrachium rosenbergii* during chilled storage. Journal of Sustainability Science and Management, 13(1), 159–167.

[fsn31706-bib-0030] Kavimandan, B. , & Saraf, M. (2016). Studies on biological efficacy of various leaf extracts of *Carica Papaya* L In International conference on global trends in engineering, technology and management (pp. 510‐516).

[fsn31706-bib-0031] Khan, M. I. , Adrees, M. N. , Arshad, M. S. , Anjum, F. M. , Jo, C. , & Sameen, A. (2015). Oxidative stability and quality characteristics of whey protein coated rohu (*Labeo rohita*) fillets. Lipids in Health and Disease, 14(1), 58 10.1186/s12944-015-0060-z 26099651 PMC4484698

[fsn31706-bib-0032] Kim, H.‐W. , Choi, Y.‐S. , Choi, J.‐H. , Kim, H.‐Y. , Hwang, K.‐E. , Song, D.‐H. , … Kim, C.‐J. (2013). Antioxidant effects of soy sauce on color stability and lipid oxidation of raw beef patties during cold storage. Meat Science, 95(3), 641–646. 10.1016/j.meatsci.2013.06.006 23811104

[fsn31706-bib-0033] Kim, I. S. , Jin, S. K. , Yang, M. R. , Chu, G. M. , Park, J. H. , Rashid, R. H. I. , … Kang, S. N. (2013). Efficacy of tomato powder as antioxidant in cooked pork patties. Asian‐Australasian Journal of Animal Sciences, 26(9), 1339 10.5713/ajas.2013.13079 25049917 PMC4093400

[fsn31706-bib-0034] Linton, M. , Mc Clements, J. M. J. , & Patterson, M. F. (2003). Changes in the microbiological quality of shellfish, brought about by treatment with high hydrostatic pressure. International journal of food science & technology, 38(6), 713–727.

[fsn31706-bib-0035] Liu, C. W. , Wang, Y. C. , Lu, H. C. , & Chiang, W. D. (2014). Optimization of ultrasound‐assisted extraction conditions for total phenols with anti‐hyperglycemic activity from *Psidium guajava* leaves. Process Biochemistry, 49(10), 1601–1605. 10.1016/j.procbio.2014.06.009

[fsn31706-bib-0036] Mailoa, M. N. , Mahendradatta, M. , Laga, A. , & Djide, N. (2014). Antimicrobial activities of tannins extract from guava leaves (*Psidium Guajava L*) on pathogens microbial. International Journal of Scientific & Technology Research, 3(1), 236–241.

[fsn31706-bib-0038] Malle, P. , & Tao, S. H. (1987). Rapid quantitative determination of trimethylamine using steam distillation. Journal of Food Protection, 50(9), 756–760. 30978808 10.4315/0362-028X-50.9.756

[fsn31706-bib-0039] Manheem, K. , Benjakul, S. , Kijroongrojana, K. , & Visessanguan, W. (2012). The effect of heating conditions on polyphenol oxidase, proteases and melanosis in pre‐cooked Pacific white shrimp during refrigerated storage. Food Chemistry, 131(4), 1370–1375. 10.1016/j.foodchem.2011.10.001

[fsn31706-bib-0041] Meilgaard, M. , Civille, G. V. , & Carr, B. T. (2007). Overall difference tests: Does a sensory difference exist between samples. Sensory Evaluation Techniques, 4, 63–104.

[fsn31706-bib-0042] Metwally, A. M. , Omar, A. A. , Ghazy, N. M. , & Harraz, F. M. (2011). Monograph of *Psidium guajava L.* leaves. Journal of Pharmaceutical Sciences, 3(21), 89–104.

[fsn31706-bib-0044] Mimica‐Dukic, N. , Bozin, B. , Sokovic, M. , & Simin, N. (2004). Antimicrobial and antioxidant activities of Melissa officinalis L. (Lamiaceae) essential oil. Journal of agricultural and food chemistry, 52(9), 2485–2489. 15113145 10.1021/jf030698a

[fsn31706-bib-0045] Mona, M. E. , Isis, A. N. , Muhammad, A. A. , & Samir, M. A. (2012). Evaluation of the biological effects for adding cinnamon volatile oil and TBHQ as antioxidant on rats' lipid profiles. Asian Journal of Plant Sciences, 11(3), 100–108. 10.3923/ajps.2012.100.108

[fsn31706-bib-0047] Nirosha, N. , & Mangalanayaki, R. (2013). Antibacterial activity of leaves and stem extract of *Carica papaya L* . IJAPBC, 2(3), 473.

[fsn31706-bib-0048] Nisar, M. F. , Arshad, M. S. , Yasin, M. , Arshad, M. U. , & Nadeem, M. T. (2019). Influence of irradiation and moringa leaf powder on the amino acid and fatty acid profiles of chicken meat stored under various packaging materials. Journal of Food Processing and Preservation, 43, e14166 10.1111/jfpp.14166

[fsn31706-bib-0049] Okoko, T. , & Ere, D. (2012). Reduction of hydrogen peroxide‐induced erythrocyte damage by *Carica papaya* leaf extract. Asian Pacific Journal of Tropical Biomedicine, 2(6), 449–453. 10.1016/S2221-1691(12)60074-4 23569948 PMC3609327

[fsn31706-bib-0051] Otsuki, N. , Dang, N. H. , Kumagai, E. , Kondo, A. , Iwata, S. , & Morimoto, C. (2010). Aqueous extract of *Carica papaya* leaves exhibits anti‐tumor activity and immunomodulatory effects. Journal of Ethnopharmacology, 127, 760–767. 10.1016/j.jep.2009.11.024 19961915

[fsn31706-bib-0052] Owoyele, B. V. , Adebukola, O. M. , Funmilayo, A. A. , & Soladoye, A. O. (2008). Anti‐inflammatory activities of ethanolic extract of *Carica papaya* leaves. Inflammopharmacology, 16, 168–173. 10.1007/s10787-008-7008-0 18759075

[fsn31706-bib-0053] Patil, T. , Patil, S. , Patil, A. , & Patil, S. (2014). *Carica papaya* leaf extracts–An Ethnomedicinal boon. International Journal of Research in Pharmacy and Chemistry, 6(2), 260–265.

[fsn31706-bib-0054] Pérez Gutiénez, R. M. , Mitchell, S. , & Vargas Solis, R. (2008). *Psidium guajava*: A review of its traditional uses, phytochemistry and pharmacology. Journal of Ethnopharmacology, 117, 1–27. 18353572 10.1016/j.jep.2008.01.025

[fsn31706-bib-0055] Prior, R. L. , Wu, X. , & Schaich, K. (2005). Standardized methods for the determination of antioxidant capacity and phenolics in foods and dietary supplements. Journal of Agricultural and Food Chemistry, 53(10), 4290–4302. 10.1021/jf0502698 15884874

[fsn31706-bib-0056] Romasi, E. , Karina, J. K. , & Parhusip, A. J. (2012). Antibacterial activity of papaya leaf extracts against pathogenic bacteria. Makara Journal of Technology, 15(2), 173–177. 10.7454/mst.v15i2.936

[fsn31706-bib-0057] Sallam, K. I. , Ishioroshi, M. , & Samejima, K. (2004). Antioxidant and antimicrobial effects of garlic in chicken sausage. LWT‐Food Science and Technology, 37(8), 849–855. 10.1016/j.lwt.2004.04.001 PMC1805705 17330154

[fsn31706-bib-0059] Schmedes, A. , & Hølmer, G. (1989). A new thiobarbituric acid (TBA) method for determining free malondialdehyde (MDA) and hydroperoxides selectively as a measure of lipid peroxidation. Journal of the American Oil Chemists' Society, 66(6), 813–817. 10.1007/BF02653674

[fsn31706-bib-0061] Sharif, M. K. , Masoos, S. B. , Hafiz, R. S. , & Muhammmad, N. (2017). Sensory evaluation and consumer acceptability. Faisalabad, Pakistan: University of Agriculture.

[fsn31706-bib-0062] Smaldone, G. , Marrone, R. , Vollano, L. , Chirollo, C. , Pellicane, A. , & Palma, G. (2011). Shelf life of thawed crustaceans treated with sulphites. Italian Journal of Food Safety, 1(1), 85–89. 10.4081/ijfs.2011.1.85

[fsn31706-bib-0063] Sofi, F. R. , Raju, C. V. , Lakshmisha, I. P. , & Singh, R. R. (2016). Antioxidant and antimicrobial properties of grape and papaya seed extracts and their application on the preservation of Indian mackerel (*Rastrelliger kanagurta*) during ice storage. Journal of Food Science and Technology, 53(1), 104–117. 10.1007/s13197-015-1983-0 26787935 PMC4711419

[fsn31706-bib-0064] Sohaib, M. , Anjum, F. M. , Arshad, M. S. , Imran, M. , Imran, A. , & Hussain, S. (2017). Oxidative stability and lipid oxidation flavoring volatiles in antioxidants treated chicken meat patties during storage. Lipids in Health and Disease, 16(1), 27 10.1186/s12944-017-0426-5 28143531 PMC5286778

[fsn31706-bib-0065] Steel, R. , & Torrie, J. (2012). Principles and procedures of statistics: A Biometrical approach MC Graw‐Hill Book Company Toronto. Revi Veteri, 13(6), 481.

[fsn31706-bib-0066] Tezcan, F. , Gültekin‐Özgüven, M. , Diken, T. , Özçelik, B. , & Erim, F. B. (2009). Antioxidant activity and total phenolic, organic acid and sugar content in commercial pomegranate juices. Food Chemistry, 115(3), 873–877. 10.1016/j.foodchem.2008.12.103

[fsn31706-bib-0067] Thuaytong, W. , & Anprung, P. (2011). Bioactive compounds and prebiotic activity in thailand‐grown red and white guava fruit (*Psidium guajava* L.). Food Science and Technology International, 17(3), 205–212. 10.1177/1082013210382066 21652766

[fsn31706-bib-0068] Udayasoorian, L. , Peter, M. , Sabina, K. , Indumathi, C. , & Muthusamy, S. (2017). Comparative evaluation on shelf life extension of MAP packed *Litopenaeus vannamei* shrimp treated with natural extracts. LWT, 77, 217–224. 10.1016/j.lwt.2016.11.046

[fsn31706-bib-0069] Verma, A. K. , Rajkumar, V. , Banerjee, R. , Biswas, S. , & Das, A. K. (2013). Guava (*Psidium guajava L.*) powder as an antioxidant dietary fibre in sheep meat nuggets. Asian‐Australasian Journal of Animal Science, 26(6), 886. 10.5713/ajas.2012.12671 PMC4093245 25049864

[fsn31706-bib-0070] Viji, P. , Venkateshwarlu, G. , Ravishankar, C. , & Gopal, T. S. (2017). Role of plant extracts as natural additives in fish and fish products‐A review. Fishery Technology, 54, 145–154.

[fsn31706-bib-0071] Vuong, Q. V. , Hirun, S. , Roach, P. D. , Bowyer, M. C. , Phillips, P. A. , & Scarlett, C. J. (2013). Effect of extraction conditions on total phenolic compounds and antioxidant activities of *Carica papaya* leaf aqueous extracts. Journal of Herbal Medicine, 3(3), 104–111. 10.1016/j.hermed.2013.04.004

[fsn31706-bib-0072] Wojdyło, A. , Oszmiański, J. , & Czemerys, R. (2007). Antioxidant activity and phenolic compounds in 32 selected herbs. Food Chemistry, 105(3), 940–949. 10.1016/j.foodchem.2007.04.038

[fsn31706-bib-0073] Yatmaz, H. A. , & Gokoglu, N. (2016). Effects of plant extract‐sulphide combinations on melanosis inhibition and quality in shrimp (*Aristeus Antennatus*). International Journal of Food Properties, 19(2), 359–370.

